# The Influence of Anti-Diabetic Drugs on Prostate Cancer

**DOI:** 10.3390/cancers13081827

**Published:** 2021-04-12

**Authors:** Miłosz Knura, Wojciech Garczorz, Adam Borek, Franciszek Drzymała, Krystian Rachwał, Kurian George, Tomasz Francuz

**Affiliations:** Department of Biochemistry, Faculty of Medical Sciences in Katowice, Medical University of Silesia, Medyków 18, 40-752 Katowice, Poland; knura.milosz@gmail.com (M.K.); adam_b1998@interia.pl (A.B.); franek175@gmail.com (F.D.); kryrach@interia.pl (K.R.); kurian.i.g@googlemail.com (K.G.); tfrancuz@sum.edu.pl (T.F.)

**Keywords:** prostate cancer, diabetes, therapy, insulin, incretin, metformin, gliflozin, thiazolidinediones, androgen deprivation therapy, metabolic pathway, DPP-4 inhibitors

## Abstract

**Simple Summary:**

A better understanding of the relation between two chronic diseases with high age-related incidence—prostate cancer (PC) and type 2 diabetes mellitus (T2DM)—seems to be crucial in a population with a growing life expectancy. In this review, a summary of the impact of widely used antidiabetic drugs on the risk of incidence and progression of PC is provided. This leads to the proposition that scientific efforts should potentially lead to the development of strategies with the most adequate treatment options of T2DM among patients with co-existing PC. Available data demonstrate that most antidiabetic drugs do not increase the risk during the treatment of patients with PC. Some reports show a potential advantage of treatment of T2DM with specific drugs. Conclusions revealed the need for further well-designed, laboratory and clinical investigations addressing the concerns raised in the issued articles.

**Abstract:**

The incidences of prostate cancer (PC) and diabetes are increasing, with a sustained trend. The occurrence of PC and type 2 diabetes mellitus (T2DM) is growing with aging. The correlation between PC occurrence and diabetes is noteworthy, as T2DM is correlated with a reduced risk of incidence of prostate cancer. Despite this reduction, diabetes mellitus increases the mortality in many cancer types, including prostate cancer. The treatment of T2DM is based on lifestyle changes and pharmacological management. Current available drugs, except insulin, are aimed at increasing insulin secretion (sulfonylureas, incretin drugs), improving insulin sensitivity (biguanides, thiazolidinediones), or increasing urinary glucose excretion (gliflozin). Comorbidities should be taken into consideration during the treatment of T2DM. This review describes currently known information about the mechanism and impact of commonly used antidiabetic drugs on the incidence and progression of PC. Outcomes of pre-clinical studies are briefly presented and their correlations with available clinical trials have also been observed. Available reports and meta-analyses demonstrate that most anti-diabetic drugs do not increase the risk during the treatment of patients with PC. However, some reports show a potential advantage of treatment of T2DM with specific drugs. Based on clinical reports, use of metformin should be considered as a therapeutic option. Moreover, anticancer properties of metformin were augmented while combined with GLP-1 analogs.

## 1. Introduction

In 2018, 1.3 million men worldwide were diagnosed with prostate cancer (PC) with an approximate mortality of 359,000. Aging and growth of the population have led to the increasing of the incidence of prostate cancer for years and this trend will continue [1]. Extrapolating epidemiological data from the past, it can be expected that prostate cancer will become a tremendous concern in the future [2]. Prostate cancer occurrence is strongly age-related (being higher in men over 65 years of age) [1]. However, some authors indicate the rising incidence of PC among young men, although the cause of this trend is so far unclear [3]. There is the hypothesis that this may be caused in part by frequent prostate-specific antigen screening, leading to overdiagnosis [3]. There is also a positive association between height, an increased level of insulin growth factor 1, and the risk of PC [4]. A small group of men with early-onset prostate cancer have a genetic predisposition, and frequently its mechanisms remain unknown. However, mutations in *BRCA1/2* and *HOXB13* genes may be associated with a higher risk of PC [5]. Suppressor genes phosphatase and tensin homolog (PTEN) play a substantial role in the pathogenesis of PC. Inactivation of PTEN by deletion or mutation is identified in approximately 20% of samples of primary prostate tumors at radical prostatectomy and in about 50% of castration-resistant prostate cancer (CRPC) [6].

Type 2 (T2DM) is the most predominant form of diabetes. Accounting for about 90% of cases, T2DM has been associated with an increased risk of developing several cancers, including liver, pancreatic, colorectal, renal, bladder, endometrial, and breast [7,8,9]. Nonetheless, what is intriguing is the fact that several meta-analyses provide evidence supporting the fact that T2DM is correlated with reduced risk of incidence of prostate cancer [10,11]. One of the possible mechanisms which explains this inverse association between diabetes and PC, is the low insulin concentration in long-term diabetes, resulting in lower plasma IGF-1 levels in diabetics compared to non-diabetics [12]. Genetic links, especially hepatocyte nuclear factor-1 β gene (HNF1β), are also considered as a potential mechanism associated with the risk of both diabetes and PC. Although, for this inverse relationship, insufficient evidence has been reported [12]. Despite this reduction, diabetes mellitus increases mortality in many cancer types, including prostate cancer [13,14].

T2DM is a complex metabolic illness characterized by hyperglycemia and progressive insulin resistance preceding its development. The prediabetes phase, along with hyperinsulinemia lasting for many years, is often associated especially with abdominal obesity [15]. Chronically elevated blood glucose leads to damage and dysfunction of various organs, particularly the heart and blood vessels, as well as retinopathy, nephropathy, and neuropathy. This disease is now a global public health problem and its incidence is increasing rapidly [16]. According to the International Diabetes Federation (IDF) in 2019, approximately 463 million adults (20–79 years) in the world suffer from diabetes and it is expected that by 2045, this will rise to 700 million [17]. Achieving glycemic control of hemoglobin A1C (HbA1C) targets of <7% and the prevention of micro- and macrovascular organ complications by individualized and optimal glycemic control is the main goal of T2DM treatment [18]. Despite the development of new medications and evidence-based treatment guidelines, a significant proportion of people with T2DM fail to achieve normal glycemic levels [19,20]. Type 2 diabetes therapy has been based on lifestyle changes and pharmacological management. Current available drugs, except insulin, are aimed at increasing insulin secretion (sulfonylureas, incretin drugs), improving insulin sensitivity (biguanides, thiazolidinediones), delaying digestion and absorption of carbohydrates from the gastrointestinal tract (α-glucosidase inhibitors) or increasing urinary glucose excretion (gliflozin) [16,21,22]. 

The androgen axis plays a crucial role in the development of PC. As a consequence of binding ligand (mostly testosterone and dihydrotestosterone) to the androgen receptor (AR), the transcription of genes engaged in the pathogenesis of PC is triggered [23]. Since the first outcomes, androgen deprivation therapy (ADT) is permanently present in therapeutical PC treatment protocols. With elapsing time of ADT, resistance to castration is developed. The underlying mechanism contains a wide array of mechanisms including modulation of receptor cofactors, AR mutations, overexpression of AR, splicing variants of AR deprived of ligand-binding domain [24]. Recently, the therapeutical use of second-generation androgen-receptor-axis-targeted agents (ARAT) like apalutamide and enzalutamide has gained growing attention, especially in the treatment of metastatic, castration-sensitive PC. Their effects were investigated in numerous clinical trials but need further assessment to be optimally used in PC therapy [25]. Results of meta-analysis added evidence that ARAT agents improve overall survival in metastatic castration-sensitive prostate cancer and discourage their combined use with docetaxel [26].

Diabetes and cancer are both associated with reduced life expectancy and their morbidity is increasing worldwide [27]. So far, in numerous meta-analyses, the inverse correlation between diabetes and prostate cancer incidence, but not mortality, has been reported. Patients suffering from PC with pre-existing diabetes had a 29% higher PC-specific mortality and a 37% higher all-cause mortality in comparison to patients presenting with PC without diabetes [28].

In addition, recent research indicates that ADT—widely used in the treatment of PC—is associated with decreased insulin sensitivity, alterations in lipid profiles, increased fat mass along with decreased lean mass. This can lead to metabolic syndrome, which occurs in >50% patients who receive long-term ADT and may contribute to non-cancer-related morbidity and mortality [29]. Furthermore, metabolic syndrome is a risk factor for the development of type 2 diabetes and cardiovascular complications [30]. The association between each of the metabolic syndrome components and benign prostate hyperplasia is supported by compelling evidence. It has been noted that the presence of at least three components of metabolic syndrome was associated with a higher risk of PC [29]. Hyperinsulinemia, an important component of metabolic syndrome enhances prostatic epithelial cell proliferation as well as PC cell plasticity, increasing tumor migration and invasiveness [31]. In the retrospective study, metabolic syndrome was responsible for shorter overall survival and shorter time to the incidence of CRPC [32]. A prospective study reveals that obesity is an independent predictor of PC. Moreover, epidemiological studies strongly suggest obesity is associated with the progression of advanced PC [33]. A long-term survival analysis of patients with PC revealed that an excess of weight (BMI > 25 kg/m^2^) leads to a four-fold higher risk of mortality independent of other clinical factors [32]. The lifestyle of elderly men has a significant impact on PC. Physical activity affects PC carcinogenesis in all stages. The molecular link between physical activity and PC includes insulin-like growth factor 1 (IGF-1), oxidative stress, inflammation, sex hormones, and myokines. Reports reveal that only about 11% of older men meet the recommendation with regards to physical activity [34]. A meta-analysis by Liu et al. found a 19% PC risk reduction for overall physical activity [35].

Up until now, there are no specific guidelines concerning modification of antidiabetic treatment among patients with co-existing PC. Clinicians should strive for optimal control of hyperglycemia which is related to an increased risk of PC recurrence [36], development of lethal PC phenotype [37]. Furthermore, choice of optimal T2DM management should be planned considering anticancer treatment to reduce its adverse effects (e.g., worse control of T2DM) and enhance its therapeutic results. Only complex and individualized therapy are possible to reduce multicausal factors of increased morbidity of PC patients with T2DM. To achieve better therapeutic effects, understanding and further investigation of the action of antidiabetic drugs is necessary.

## 2. Insulin

Patients with diabetes type 2 usually are insulin resistant and the blood level of insulin also depends on the stage of diabetes. Although metformin is the recommended primary treatment of type 2 diabetes, the role of insulin cannot be underestimated [38]. The use of insulin is indicated in the case of ineffective glycemic control, with HbA1C levels over 9% [75 mmol/mol] and presented symptoms of hyperglycemia. Insulin is the most efficient hypoglycemic pharmacological agent, and its functions include suppressing hepatic gluconeogenesis and glycogenolysis, while stimulating glucose uptake into muscle and adipocyte tissues. Furthermore, insulin’s role is to inhibit lipolysis which results in reducing plasma-free fatty acids levels [38,39]. Insulin resistance is defined as the inadequate response of peripheral tissues to insulin. This condition is broadly described not only in T2DM but also in obesity, dyslipidemia, and cardiovascular diseases [40]. As a reaction to the mentioned insulin refractory, hyperinsulinemia occurs. Elevated concentrations of C-peptide in the fasting state have also been associated with PC risk and higher-grade cancer. C-peptide serum concentration reflects insulin secretion as a reliable measurement, because it is not metabolized by the liver [41]. Leptin and adiponectin may also be involved in the relationship between PC and obesity, especially in the development of insulin resistance. Leptin is elevated in obese men, and there are reports connecting leptin with increased prostate cancer cell proliferation and inhibition of apoptosis. Adiponectin has anti-tumorigenic effects, but in contrast to leptin, its concentration in serum is reduced in obese people. There is an inverse correlation between histological grade, and disease stage, and plasma adiponectin levels in patients with PC [41].

**Figure 1:**
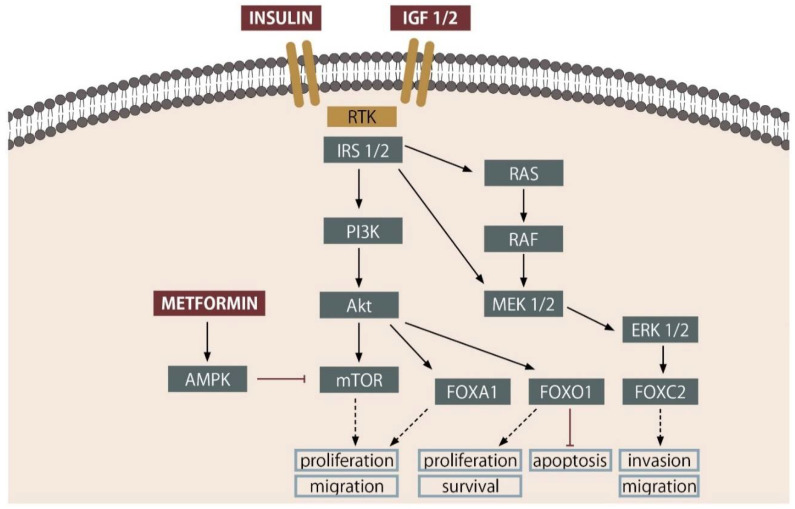
Influence of insulin and metformin on PC cells. Insulin in PC cells activates both MAPK/Erk1-2 and PI3K-Akt signaling pathways. mTOR and FOX-family proteins play a crucial role in the proliferation, migration and invasion. (Detailed description in text). RTK—Receptor tyrosine kinase, Akt—protein kinase B, IRS—Insulin Receptor Substrate Proteins, PI3K—Phosphoinositide 3-kinase, MEK—Mitogen-activated protein kinase kinase, RAS/RAF—Serine/threonine-specific protein kinases, ERK—extracellular signal-regulated kinases, mTOR—mechanistic target of rapamycin, AMPK—5’AMP-activated protein kinase, FOX—forkhead box proteins.

Besides systemic regulation of metabolic homeostasis, insulin can act as a cellular growth and proliferation factor. Signaling pathways activated by insulin are altered in cancer cells, including prostate cancer. This suggests the potential influence of insulin as a pro-tumorigenic factor. Binding to its receptor on the cell surface, insulin initiates a cascade of events inside the cell. The beginning of this process is the activation of the protein-tyrosine kinase domain, which causes the autophosphorylation of the insulin receptor substrate 1 and 2 (IRS1 and IRS2). This directs the signal to the phosphoinositide 3-kinase (PI3K)–Akt and mitogen-activated protein kinase (MAPK) signaling pathway—Figure 1 [42,43]. The PI3K-signalling cascade coordinates systemic nutrient status with cellular activities such as the intake and utilization of glucose, protein synthesis, and the cells’ growth [44]. It also responds to extracellular signals other than insulin, such as peptide hormones, epidermal growth factor (EGF), or insulin-like growth factor 1 (IGF-1). The expression of IGF1-R in the tumor may play a role in prostate cancer progression to a lethal phenotype, which may be more sensitive to IGF signaling. Roughly 40% of primary and 70% of metastatic prostate cancers have genomic alterations in the PI3K signaling pathway, mainly as a result of PTEN loss. One of the key downstream effects of PI3K is the serine/threonine kinase AKT, which phosphorylates Forkhead Box Protein (FOX) and induces its translocation from the nucleus to the cytoplasm. This prevents FOXO1 from acting as a transcriptional regulator in inhibition of ligand-induced AR activation. Similarly, in PTEN-negative prostate cancer, PI3K/AKT signaling is activated and the downregulation of FOXO1 contributes to the hyperactivation of AR, thereby driving the castration-resistant progression of PC [42].

Researchers indicate the FOX protein family as a connector between the insulin/IGF-1 and androgen signaling pathway. The O-class of FOX proteins is a corepressor of androgen receptor (AR) activation, whereas A-class contributes to transactivation of AR and attenuates PC growth. Moreover, FOXO1 is also engaged in taxane-mediated (docetaxel is the first-line chemotherapy in castration-resistant PC) attenuation of androgen receptor activation and progression to CRPC [43]. In another in vitro study, insulin via the upregulation of Forkhead Box Protein C2 (FOXC2) promotes migration and invasion of prostate cancer cells and activation of insulin signaling pathway and inhibition of PI3K and MAPK pathways lead to epithelial-mesenchymal transition [44].

A more abundant expression of insulin receptors in malignant than benign prostate epithelial cells was observed via immunoenzymatic staining. The results prompt the authors to conclude about the crucial role of insulin level in the pathogenesis and progression of PC [45]. Additionally, immunoenzymatic staining of PC tissue revealed increased expression of IGF-1 receptors in high-grade specimens than in low grade [46]. Additionally, in the work of Ahearn et al. a higher expression of IGF-1 receptors was proved in PC with increased cell proliferation, whereas a more intensified response of IGF-1 signaling pathway is shown in erythroblast transformation-specific related gene (ERG) positive than in ERG-negative tumors. No correlation between the concentration of insulin and the progression of PC was observed. These results confirmed the statement that IGF receptors are likely to be engaged in PC carcinogenesis [47].

Some pre-clinical evidence indicates long-acting insulin-glargine exerts more pro-cancer effects than other analogs. This possible action was hypothesized because of in vitro experiments that identified higher affinity of insulin glargine to IGF-1 receptors than human insulin [48,49].

A systematic review and meta-analysis of Karlstad et al. took into consideration the risk of PC in three groups. The observed PC incidence rate among insulin-users (at least 5 years) in comparison to non-users was lower in the insulin-user group (RR 0.80 95% CI: 0.73–0.88). While comparing insulin-users to users of other anti-diabetic drugs and more specifically, glargine-users to non-glargine users, there were no statistically significant differences between analyzed subpopulations [50]. Some cohort studies display no association between PC risk with glargine and non-glargine insulin users. Other meta-analyses, however, indicate that insulin lowers PC risk but glargine users have the same risk compared to non-glargine users (OR 0.94, 95% CI 0.63–1.42) [51]. Meta-analysis performed on a total of 1143 men in the prostate cancer group and 1692 men in the control group has shown that patients with prostate cancer had insulin level significantly higher in comparison with the control group. Sub-group analysis has pointed out that this correlation takes place only in older patients (>65 years old) [52]. In a prospective study examining 310 patients (54 of them died because of PC during 5-years follow-up), hyperinsulinemia and T2DM have been shown to have a statistically significant correlation with lethal prostate cancer [53]. Up to now the most complex meta-analysis of 205,523 male subjects and 7053 PC cases demonstrate no association between PC risk and insulin use, in comparison to other antidiabetic drugs (RR = 0.89, 95% CI: 0.72–1.09). There is no significant evidence that glargine-insulin users have higher PC risk than non-glargine insulin users (RR 1.26, 95% CI: 0.86–1.84) [54].

## 3. Sulfonyloureas

Sulfonylureas are a heterogeneous group of antidiabetic drugs that prompt increased insulin secretion. The mechanism of action is exerted by the closing of ATP-dependent potassium channels in the β-pancreatic cell membrane which leads to its depolarization and triggers the opening of voltage-gated Ca^2+^ channels. The calcium ions are the direct stimulus for the exocytosis of insulin [55].

Until now, relatively few results concerning the impact of SU on PC cells in vitro and in vivo were published. Prostate cell lines treated with glibenclamide–second-generation sulfonylureas display in vitro dose-dependent inhibition of growth and cellular apoptosis in concentration 0.1 mM (50 µg/mL) [56]. In another study, glipizide (5 mg/kg) in the murine model of PC suppresses angiogenesis, but not cell proliferation [57].

Results of the Finnish randomized study reveal that among investigated groups of antidiabetic medication, only SU increases the risk of metastatic PC (HR = 2.04, 95% CI 1.11–3.77). That indirectly suggests that hyperinsulinemia may be a risk factor for PC, and thus indicates insulin secretion inducing features of SU as a main cancerogenic factor [58]. In a Japanese population-based retrospective study of 121 patients with metastatic prostate cancer treated with primary ADT with castration and/or an antiandrogen agent (bicalutamide or flutamide), sulfonylurea treatment was associated with longer progression-free survival (PFS) in men with diabetes mellitus. In addition, diabetes, but not dyslipidemia has possible adverse prognostic factors for overall survival (OS). This study had several limitations including a small sample size, retrospective research design, absence of information on either serum glucose or lipid level in a small fraction of the cohort, long accrual period between 2001 and 2013 (before some new drugs were introduced). Moreover, this study included a homogenous study population. Consequently, further investigation will be necessary [59].

## 4. Metformin

Metformin (Met) is the drug of first choice in type 2 diabetes mellitus. It reduces the level of circulating glucose and is particularly effective against insulin resistance and in obese patients. In the animal models, metformin inhibited proliferation of tumor cells, but not cell migration of PC [60]. Using metformin also induces apoptosis via activation of AMPK (AMP-activated kinase) pathway in prostate cancer cells [60]. AMPK is a regulator sensitive to cell energy status, it controls the balance between the anabolic and catabolic processes. Through enzyme phosphorylation and regulation of gene expression, it allows cells to adapt to environmental conditions [61]. Inhibiting proliferation is also reached by blocking the cell cycle in G0/G1. Metformin decreases cyclin D1 level, pRb phosphorylation, and increases p27kip protein expression [62]. Metformin also is effective in lowering IGF-1 and insulin levels. These hormones can stimulate prostate cancer proliferation through activation of the FOXO1 subunit of the androgen receptor [63]. Metformin upregulates REDD1 (regulated in development and DNA response-1) that promotes cell cycle arrest and inhibits PI3K/AKT/mTOR (Figure 1). These actions lead to tumor suppression and increase apoptosis [64]. Met also inhibits NF-κB, leading to delay of cell aging. However, modulation of inflammatory cytokines profile leads to improved response against cancer cells [65,66].

Despite the promising outcomes of the wide array of pre-clinical studies, clinical trials considering the risk of PC incidence and progression of this malignancy present with varying results upon administration of Met. The available data present a spectrum of findings of Met having reduction of risk [58,67] no effect [68], to even an increased risk of PC [69]. Similar discrepancy is observed in meta-analyses. In the works of Yu and Deng, statistically significant reduction of PC risk was associated with metformin therapy [70,71]. These two meta-analyses, Yu et al. from 2014 and Deng et al. from 2015, are based on older observational studies, and consequently, less patients are included. The outcome of the Ghiasi meta-analysis also indicates lower PC incidence, although it was not statistically significant [72]. In this meta-analysis, only 11 observational studies were included. However, recent three large meta-analyses [73,74,75] revealed no association between metformin use and the risk of prostate cancer. Particularly, He et al. in a meta-analysis from 2019 included 1,660,795 patients and Wang et al. in a meta-analysis from 2020 included 2,009,504 patients, which makes these analyses the largest so far [74,75]. The work of Chen focuses on the possible influence of race on the effect of metformin on PC occurrence did not show any differences between the Asian and so-called Western populations. Moreover, the correlation was not proved in both groups [76]. All details and results of mentioned meta-analyses are presented in Supplementary Table S1.

Another aspect that should be taken into consideration in clinical studies is the impact of metformin on the progression of disease among patients with already diagnosed PC and further therapy outcomes. Some previous research articles by Raval et al. and Deng et al. do not support a beneficial correlation between all-cause mortality and metformin use. As well as no association with cancer-specific mortality and metastasis, there is no supporting evidence of a positive impact on the recurrence of PC [71,77]. In the results of all meta-analyses from the last 5 years, published by He et al. in 2019, Coyle et al. in 2016, Xiao et al. in 2017, and Stopsack et al. in 2016, overall survival among patients with PC treated with metformin was improved [74,78,79,80]. Also, the recurrence of PC among metformin-users in the recent three large meta-analyses is supposed to be decreased [74,78,81]. These meta-analyses included a larger patient database than older ones. The mentioned research articles use different survival analysis statistics. The reason for the discrepancy among presented studies could be confounding factors and heterogeneity between research samples. The results are presented in Supplementary Table S1.

The variance between some of the presented works is due to many factors impeding the design of studies, such as the underlying of T2DM and other diseases, duration of treatment, and cumulative and dose-dependent effect, different ways of heterogeneity assessment of included in meta-analyses. Considering that, many authors and clinicians emphasize an urge to conduct well-designed prospective large-sample randomized studies.

However, the impact of Met on PC was broadly discussed in several reliable reviews and present compelling results about the potential use of metformin in the systemic treatment of PC [82,83,84] but with some co-existing limitations. Patients with diabetes mellitus have a lower incidence of prostate cancer due to low androgen, insulin, and IGF-1 levels [84]. The dose of metformin used in in vitro studies when extrapolated to human organism exceeds level far beyond metformin tolerance. On the other hand, decrease in dose could not develop a desirable anti-cancer effect. The average level of metformin in plasma of diabetic patients reaches 10 µM, with the maximum of 40 µM after standard doses [85]. Moreover, levels that should be reached to obtain biological effect in preclinical studies conducted on PC lines fluctuate from 1 to 30 mM [82,86]. The genetic and biological heterogeneity of PC determines the efficacy of metformin. The sustained presence of micromolar-concentration of metformin may be responsible for these cumulative biological effects.

The results of a multi-arm multi-stage randomized controlled trial called STAMPEDE will provide precise and necessary information about additive use of tested drugs in the systemic treatment of PC. The planned finish of the trial is estimated to be in 2024 and one of the trial’s arms commenced in 2016 investigate the action of Met. Based on the fact that this is a large group with a prospective and well-designed character of study, the results to be received will be of utmost importance for the understanding of Met and its effects [87]. Another interestingly designed trial called—METAL (metformin and longevity) is a randomized, placebo-controlled, double-blind study, aiming to identify molecular mechanisms underlying the effect of metformin on PC. The authors are planning to examine 100 patients with PC before prostatectomy in two groups—metformin users and placebo [88]. Previous studies in this area were retrospective or not focused on prostate cancer. Therefore, only new, prospective studies may provide conclusive results on the effect of metformin on the progression of PC.

## 5. DPP-4 Inhibitors

Dipeptidyl peptidase 4 (DPP-4) gene encodes a membrane-anchored protein that cleaves dipeptides from multiple substrates, resulting in their increased degradation and thus regulates the activity of a wide array of peptides, for example: incretins, chemokines, neuropeptides [89]. It is especially concerning incretins–glucagon-like peptide-1 (GLP-1) and glucose-dependent insulinotropic polypeptide (GIP), described below.

Multiway regulatory function of DPP-4 impels to hypothesis about its possible suppressing or promoting impact on cancers including prostate cancer. Dipeptidyl peptidase 4 glycoprotein is found to be present in the prostate, more abundantly in the transition (TZ) than the peripheral zone (PZ). However, in prostate cancer tissue expression of DPP-4 is significantly doubled, with inversion of TZ/PZ ratio [90]. Early cell lines and animal studies on prostate cancer showed that increased expression of DPP-4 may play an important role as a factor suppressing the long-period progression of the disease [91]. But, recent results of clinical trials showed the opposite [92,93]. Such contrary results may be due to the activity of DPP-4 on many substrates, which were not present, nor taken into consideration during cell line studies. Moreover, some experimental studies of DPP-4 inhibition used diprotin—a tripeptide significantly differ from gliptin drugs—which are not of peptide structure. DPP-4 is known for proteolysis of much more chemokines [94]. An example being the proteolytic cleavage of CXCL10 by DPP-4, which leads to decreased migration of T-lymphocytes, notably, those with the CXCR3 receptor expressed on their surfaces. This process contributes to the suppression of the immune response within a tumor environment. Authors suggest that inhibition of DPP-4 enhance immunomodulatory properties and in the future could be used as an adjuvant to PC immunotherapy [95].

A study by Nazarian et al. had shown a decreased level of DPP-4 transcript in PC animal models (mice c-Myc, knock-out p53, and PTEN) as well as, in the serum samples gathered from patients with PC (*n* = 144) in comparison to the control group. Furthermore, DPP-4 activity was lower among patients with a metastatic disease [96]. The involvement of DPP-4 in metastasis of prostate cancer cells was shown in the work undertaken by Sun et al. The experiments showed (both in vitro and in vivo) that proteolytic cleavage of CXCL12 by DPP-4 protects from finding to its receptors CXCR4 and CXCR7/RDC1 and thus protect from neoplasm invasion. Therefore, its inhibition leads to the reverse of the observations [97].

In the work of Russo et al. on the murine xenograft model with VCaP, cancer cells attenuated expression of DPP-4 while a progression to CRPC. Results from animal models were confirmed in the immunohistochemical staining of clinical samples, where the quantity of DPP-4 protein from patients with CRPC was also decreased. Attempts to restore DPP-4 expression by testosterone injection of investigated mice subjects revealed that DPP-4 downregulation was due to an epigenetic reversible mechanism. Results presented in this study prove that DPP-4 functions as an androgen receptor-stimulated tumor suppressor gene and indicate that treatment with gliptins could aggravate treatment of PC based on ADT [98].

So far, not many population studies were conducted regarding the impact of DPP-4 on PC progression. In a retrospective cohort of Taiwanese men population with newly diagnosed T2DM subjects and controls recruited from the National Health Insurance database in years 1999–2010, sitagliptin demonstrates its beneficial effect on the risk of PC. In a group of diabetic men treated with sitagliptin (*n* = 37,924) incidence of PC was significantly lower in comparison to sitagliptin never-users (*n* = 426,276) (HR = 0.517 (95% CI: 0.339–0.788) [92], a period of drug cumulation > 12.7 months and cumulative dose > 33,600 mg. Another retrospective observational study assessed risk of PC metastasis according to data from Disease Analyzer—a medical database gathering information from German general practitioners and internal medicine practices. Analysis of 906 subjects: 453 with metastatic PC and matched same-number control group (based on propensity score matching) displayed no coincidence between the development of prostate cancer metastases and DPP-4 inhibitor use [99]. Recent retrospective cohort analysis of 15,330 patients with PC and diabetes showed that use of DPP-4 inhibitors only (*n* = 441) (HR 0.77; 95% CI: 0.64–0.93) and in combination with metformin (*n* = 820) (HR 0.80; 95% CI: 0.68–0.94) had a significant overall survival (OS) benefit compared to the reference group (not on either DPP4 inhibitors or metformin). In addition, DPP-4 inhibitors show a trend to bring neutral or favorable results in subgroup analyses of PC patients irrespective of the stage (stage I not reliable; stage II HR 0.70, 95% CI: 0.54–0.9; stage III HR 0.96, 95% CI: 0.26–3.55; stage IV HR 0,81 95% CI: 0.53–1.26), treatments with chemotherapy (HR 0.84, 95% CI: 0.65–1.08 with chemotherapy and HR 0.71, 95% CI: 0.53–0.95) without chemotherapy), androgen-deprivation therapy (ADT) (HR 0.87, 95% CI: 0.69–1.15 with ADT and HR 0.67, 95% CI: 0.5–0.84 without ADT), prostatectomy (HR 0.27, 95% CI: 0.04–2.00 with prostatectomy and HR 0.78, 95% CI: 0.65–0.95 with no prostatectomy), or radiation (HR 0.84, 95% CI: 0.59–1.19 with radiation therapy and HR 0.73, 95% CI: 0.58–0.91 without radiation therapy), however because of low sample size some of this data was not statistically significant [93].

## 6. Incretins

The two most important natural incretin hormones are glucagon-like peptide-1 (GLP-1) and glucose-dependent insulinotropic polypeptide (GIP), both acting on pancreatic β cells through their respective receptors. However, GIP and GLP-1 are not used as drugs, due to their rapid hydrolysis by DPP-4 which reduces their lifespan up to only 1–3 min [100]. Consequently, current pharmacological approaches are focused on the use of GLP-1 analogs resistant to DPP-4 enzymatic degradation such as liraglutide and exenatide. GLP-1 receptors are present in pancreatic islets, stomach, heart, brain, and kidney. GLP-1 is responsible for the regulation of appetite and food intake. In the pancreas, it stimulates insulin and somatostatin secretion, whereas strongly inhibit glucagon release [101].

Due to the results of animal studies of incretin drugs, there was some concern that they may increase the risk of malignant neoplasia [102]. Nachnani et al. have shown that exenatide, a GLP-1 receptor agonist, promotes pancreatic duct hyperplasia in rats [103]. In preclinical studies, the incidence of thyroid C-cell tumors was increased in rodents treated with GLP-1 analogs [104]. Moreover, there were clinical reports suggesting that GLP-1 receptor agonists are associated with an increased risk of pancreatitis, a known risk factor for pancreatic cancer [102]. Two contemporary systematic reviews found no association between the use of GLP-1 analogs and the incidence of neoplasia in general [105,106]. The LEADER trial (Liraglutide Effect and Action in Diabetes: Evaluation of Cardiovascular Outcome Results) is a randomized, double-blind, controlled trial in which a secondary outcome incidence of prostate cancer was lower in the group of liraglutide-user (*n* = 26) in comparison to placebo (*n* = 24) with HR 0.54 (95% CI: 0.34–0.88). As the study was not primarily aimed to assess neoplasia occurrence authors emphasize the urge of further large sample prospective studies [107]. So far, there is no evidence of how treatment with GLP-1 analogs could influence PC management.

**Figure 2:**
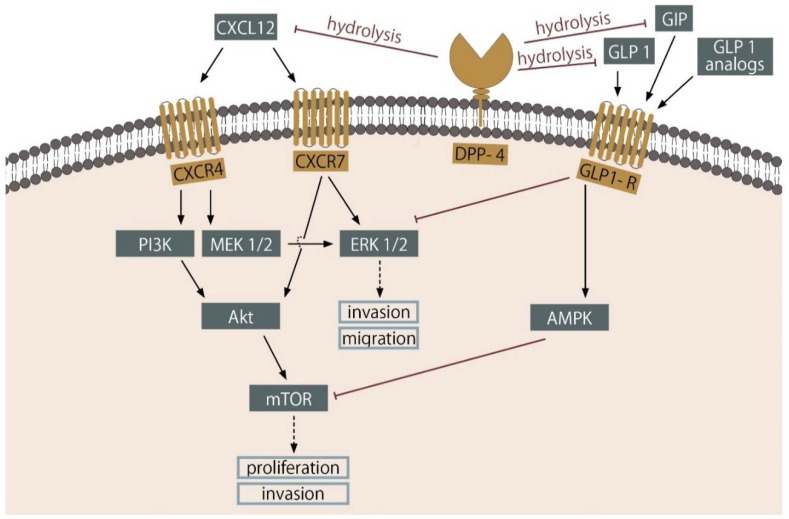
Mechanisms of GLP-1 and DPP-4 action on PC cells. In vivo experiments revealed that GLP-1 analogs inhibit signaling pathways engaged in tumorigenesis of PC. DPP-4 decreases the level of functional GLP-1 and GIP by its hydrolysis, and through this, attenuates binding of CXCL-12 to receptors CXCR4 and CXCR7 (detailed description in text). CXCL—ligand of chemokine receptor, PI3K—Phosphoinositide 3-kinase, Akt—protein kinase B, MEK—Mitogen-activated protein kinase kinase, ERK—extracellular signal-regulated kinases, mTOR—mechanistic target of rapamycin, AMPK—5’AMP-activated protein kinase.

Nomiyama et al. identified that human prostate cancer tissue expresses a large amount of glucagon-like peptide-1 receptor. Research performed on the cell lines LNCap, ALVA-41 (androgen-sensitive prostate cancer) and PC3 (DU145 androgen-independent prostate cancer) demonstrated that GLP-1R mRNA was abundantly expressed in LNCap and DU145 cells, but was significantly lower in PC3 and ALVA-41 cells. Therapy with Ex-4 relevantly reduces the proliferation of LNCap, DU145, and PC3 but does not induce apoptosis. The most significant reduction of proliferation was observed in LNCap. It may be presumably due to the highest GLP-1R expression. The GLP-1R antagonist and the protein kinase A inhibitor (PKI) canceled the antiproliferative effect of Ex-4, which suggests that the Ex-4 reduces prostate cancer cell proliferation due to GLP-1R activation which results in inhibition of ERK-MAPK. LNCap cells were transplanted into athymic mice, and the therapy with Ex-4 significantly reduce the size of tumor [108]. In another study, researchers investigated the relationship between the expression of GLP-1 receptors and the development of PC. In the 30 human samples of PC GLP-1 receptor expression was inversely associated with cancer progression (Gleason score). Additionally in vitro and in vivo antiproliferative effects of GLP-1 receptors in ALVA-41 cells were evaluated and revealed that the presence of GLP-1 receptors inhibits PC cell proliferation by suppressing cell cycle progression [109]. Furthermore, the study of He and Li reveals that Exendin-4 sensitizes prostate cancer cells to radiation. Since radiotherapy is an important treatment for prostate cancer, the influence of such drugs used in comorbidities may impact the PC outcome. Investigated GLP-1 analog may act through enhancement of AMPK phosphorylation and diminishment of mTOR/cyclin B/p34 expression [110]. Recent research carried out on LNCap and CWR22RV1 cells transplanted into mice and above cell lines in vitro, have shown that therapy with enzalutamide and Ex-4 in combination is prominently more effective than either medicine alone [111]. Exendin-4 is able to antagonize enzalutamide-induced invasion and migration of both prostate cancer cells. It is indeed essential, that these results from the animal model must be confirmed by further clinical trials. Presented results are molecularly caused by exendin-4 inhibition of PI3/Akt/mTOR signaling pathway which activation leads to androgen refractory (Figure 2). Moreover, the mentioned drugs combination decrease expression of androgen-receptor splice variant 7 (AR-V7) and nuclear localization of full-length androgen receptor [111]. In the recent preclinical study, liraglutide was revealed to act synergistically with docetaxel on the attenuation of LNCaP—prostate cancer cell growth. Co-administration of these two agents inhibits both ERK-MAPK and PI3K/AKT pathways, moreover PC cell cycle arrest in phase G2/M was observed [112].

## 7. SGLT2 Inhibitors

Tumor cells have an increased demand for glucose uptake to fuel ATP synthesis compared to normal cells. This is done through the upregulation of specific transporters: the facilitated diffusion glucose transporters (GLUTs) superfamily (SLC2A) and sodium-glucose linked transporter (SGLTs) family (SLC5). The overexpression of GLUTs, mainly GLUT1 has been shown in many cancer types. Later studies have shown expression of SGLT2 in, among others, pancreatic, lung, brain, and prostate cancer [113].

Dapagliflozin, canagliflozin, empagliflozin are inhibitors of sodium-dependent glucose co-transporter 2 (SGLT2). They have been registered for the treatment of type 2 diabetes by the European Medicines Agency (EMA) and the Food and Drug Administration (FDA) [114]. They competently, reversibly and highly selectively block SGLT2 located in the proximal nephron tubules, which are responsible for the resorption of approximately 90% of urinary glucose. As a consequence, glucose is excreted in the urine and its plasma concentration is normalized in an insulin-independent mechanism [114,115]. Scagoflio et al. in their work, based on immunohistochemical tests using specific antibodies, concluded that both SGLT1 and SGLT2 are expressed in human prostate adenocarcinoma. In tumor regions with normal histology, SGLT1 was expressed in prostate ducts, but SGLT2 was not detected. In tumors obtained from patients, they detected SGLT2 expression in regions with high uptake of an SGLT-specific radioactive glucose analog, α-methyl-4-deoxy-4-[18F] fluoroglucopyranoside (Me4FDG), and its functional activity was blocked by specific SGLT inhibitors (phlorizin or dapagliflozin). Both phlorizin and dapagliflozin reduced malignant tissue uptake of Me4FDG. Similar results were obtained during in vivo studies in PC-3 mouse xenograft models of prostate cancer in which Me4FDG uptake was blocked by dapagliflozin. They also provided preliminary evidence on the efficacy of SGLT2 inhibitors in reducing growth and/or increasing tumor necrosis in the pancreatic xenograft model [116]. It has been reported that SGLT1 may strongly interact with epidermal growth factor receptor (EGFR), which is overexpressed in more than 80% of the late stages of prostate cancer and is associated with poor prognosis. EGFR, by interacting with SGLT1, is involved in maintaining basal intracellular glucose levels in cancer cells. They showed that inhibition of SGLT1 by its inhibitor (florinsine) sensitized prostate cancer cells to treatment with an EGFR tyrosine kinase inhibitor (gefitinib and erlotinib) [117].

In vitro studies showed that clinically achievable concentrations of canagliflozin (range from 5 to 30 µM) inhibited the proliferation and clonogenic survival of prostate cancer cells (PC3 and 22RV-1). In combination with docetaxel as well as with ionizing radiation, canagliflozin improved their effectiveness. In contrast, dapagliflozin did not demonstrate such properties. In PC3 cell line canagliflozin, but not dapagliflozin reduced the uptake of 2-deoxy-D-glucose in a dose-dependent manner. The antiproliferative effect of canagliflozin was similar, and independent of glucose concentration, suggesting that the attenuation of glucose uptake was not the primary factor limiting cell growth. It has been shown that canagliflozin (30 µM) has the ability to rapidly activate AMPK, which resulted in inhibiting lipogenesis through acetyl-CoA carboxylase (ACC) phosphorylation. This was confirmed by observations in PC3 xenografts of mice and in vitro studies. However, this effect was not necessary for antiproliferative activity, because both overexpressing a dominant-negative AMPK or blocking AMPK functions did not affect canagliflozin’s ability to reduce cell proliferation. In this study, dapagliflozin did not show any of the above effects reported for canagliflozin [118].

During early clinical studies, alarming data on the potentially increased incidence of breast cancer in women and bladder cancer in men was evident during dapagliflozin administration, which initially hindered its approval by the FDA in 2012. However, these data were based on the short-term study and were not statistically significant [119]. In 2017, meta-analysis of 580 cancer cases among 34,569 participants from 46 short-term randomized controlled trials (RCTs) indicated that treatment with SGLT2 inhibitors was not significantly associated with an increased risk of overall cancer. However, it was noted that SGLT2 inhibitors were associated with an increased risk of overall cancer among obese participants (BMI ≥ 30 kg/m^2^) [120].

The meta-analysis from 2019 by Dicembrini et al. is the biggest to date about SGLT2 inhibitors and the incidence of cancer includes 48,185 patients, among them 27,744 patients in the SGLT2 group. All included trials lasted more than 52 weeks. There was no difference in the incidence of all malignancies. In this meta-analysis, the incidence of PC has the Odds Ratio of 0.97 (95% CI: 0.61–1.52). The duration of the mentioned meta-analyses is the most important limitation, because potential effects of treatment may develop in a long time [121].

## 8. Thiazolidinediones

Peroxisome proliferator-activated receptors (PPARs) are a group of nuclear receptor proteins playing the role of ligand-activated transcription factors. There are distinguished three isoforms of PPARs: alpha (α), beta/delta (β/δ) and gamma (γ) and differs from each other in expression in human tissues and specific regulatory function of metabolism. Thiazolidinediones (TZDs) are activators of isoform gamma and thus enhance insulin sensitivity and regulate adipogenesis in a way that allows them to be used in T2DM treatment [122]. Researchers draw attention to the existence of two PPARγ 1 and 2 varying by 30 amino acids and their function in PC tumorigenesis. Data collected from the study conducted on PC cell lines suggest that PPARγ1 enhances PC cell proliferation and the transformation of benign prostate epithelial cells, whereas PPARγ2 attenuates PC cells growth [123]. 

The inhibitory effect of pioglitazone on PC cell lines exerted during in vitro studies was mediated by suppression of cyclin D1 expression and the activation of p38 MAPK and NFκB pathway. These molecular mechanisms were also confirmed in vivo using a transgenic rat with adenocarcinoma of prostate (TRAP) model treated with TZD. The number of PC lesions and Ki67 labeling index were decreased [124]. A PPARγ-dependent signaling pathway is mediated also by the upregulation of E-cadherin and glutathione peroxidase 3 which are known for their role in tumor invasion and migration [125]. TZD act also in PPAR-independent mechanisms and pathways. PPARγ agonists attenuate the growth of PC cells by inhibition of expression of vascular endothelial growth factor (VEGF) and phosphorylation of AKT [126] and as well suppression of C-X-C chemokine receptor type 4/C-X-C motif chemokine 12 (CXCR4/CXCL12) axis and Bcl-xL/Bcl-2 function [127]. PPARγ and testicular receptor 4 (TR4, NR2C2) belong to a wide array of nuclear receptors. TZD drugs were designed as ligands to PPARγ transactivate TR4. In one study it was shown that knock-down of TR4 enhance PC progression in vivo and thus concluded that TZD impact on PC cells depends on TR4 expression in PC cells and could cause various side-effect among the treated individuals [128]. Additional reports reveal that TR4 promotes metastasis [129] and enhances the molecular response of PC cells to radiotherapy and chemotherapy [130]. The role of the androgen receptor signaling pathway in the carcinogenesis of PC is supposed to be crucial. Some reports indicate signaling crosstalk between androgen and PPAR pathways. PPAR ligands can activate or inhibit androgen signaling depending on PC resistance to ADT. Contrarily, activation of androgen receptors decreases PPAR activity. Considering that investigating the mechanisms of PPAR involvement, could contribute to a better understanding of PC biology [131,132].

Clinical data considering the incidence of prostate cancer among pioglitazone users are not consistent. PROactive’ study an observational follow-up reveals that prostate cancer seems to occur more frequently in the group of pioglitazone-users in comparison to placebo [133]. In a study with the main aim to investigate the impact of pioglitazone on bladder cancer, in a group of 3777 patients with prostate cancer, the incidence of PC was higher in a group of pioglitazone users than non-users (HR 1.13; 95% CI: 1.02–1.26) [134]. On the other hand, placebo double-blind study IRIS, represents a different perspective and does not support the idea of diverse PC prevalence in TZD-user and placebo group [135], as it showed no observable difference. In another Taiwanese population-based (3513 patients with PC aged over 40 years, among them 178 treated with PGZ and one control subject per case) no association between prior pioglitazone usage and prostate cancer occurrence was demonstrated [136].

## 9. Conclusions and Further Perspectives

Due to the increase in predicted average lifetime and incidence of diabetes, it can be expected that an increased focus on the efficacy of treatment of prostate cancer, as well as the quality of life of such patients will result. The co-existence of these two chronic diseases may require some modifications in the treatment of diabetes. This review aimed to investigate currently known information about the impact of commonly used antidiabetic drugs on the incidence and progression of PC. Crawley and co-authors of a systematic review emphasize the complex relationship between PC and T2DM, not only in terms of the prevalent co-existence but also their impact on the course of the disease, mortality, and interaction between T2DM and PC management [86]. We briefly investigated the outcomes of pre-clinical studies and we looked for its correlation with available clinical trials (the outcomes of pre-clinical studies and their correlation with available clinical trials were briefly investigated (discussed)). Available reports and meta-analyses demonstrate that most anti-diabetic drugs do not increase the risk during the treatment of patients with PC. However, some reports show a potential advantage of T2DM treatment with specific drugs, particularly in the light of the recent reports about metformin as a possible therapeutic option. The effect of other widely used anti-diabetic drugs on PC should be considered. Moreover, in the study of Tsutsumi and co-researchers, Met anticancer properties were augmented while combined with the GLP-1 analog exendin-4 [60].

Many available anti-diabetic drugs with different mechanisms of action require a rational decision based on scientific evidence and clinical trials to choose the best option for the patient with concomitant PC. Further interest in antidiabetic drugs and their impact on PC should be considered in terms of:

- stratification of the risk of PC related to the treatment of T2DM,
- optimization of T2DM treatment among patients with PC (concerning the metabolic effect of ADT),
- possible use of antidiabetic drugs in the management of PC in non-diabetic patients.

Results of clinical trials and meta-analyses are limited, especially for more modern drugs like DPP-4 inhibitors, incretin agonists, and SGLT2 inhibitors. Therefore, we emphasize an urge to conduct further well-designed both pre-clinical and clinical studies concerning new antidiabetic drugs.

## Supplementary Materials

The following are available online at https://www.mdpi.com/article/10.3390/cancers13081827/s1, Table S1: Recent meta-analysis concerning the use of metformin and risk and progression of PC.
