# Supplementary material for: The Influence of Anti-Diabetic Drugs on Prostate Cancer
Source: Cancers (Basel). 2021 Apr 12;13(8):1827. doi: 10.3390/cancers13081827 (PMC8068793; doi:10.3390/cancers13081827)
Supplement: Supplementary file 1 [file cancers-13-01827-s001.pdf]

Supplementary Table 1. Recent meta-analysis concerning the use of metformin and risk and progression of PC.
HR - hazard ratio, pHR- polled hazard ratio, RR - relative risk, OR - odds ratio, 95% CI - 95 % confidence interval, P - p-value, OS - overall survival, CSS - cancer-specific survival, ACM- all cause mortality, CSM- cancer-specific mortality.

First author / Year of publication	Sample size	No of studies included	Result/ conclusion	Risk of PC incidence	Overall survival	Cancer-specific survival	Recurrence-free survival	Risk of recurrence	All-cause mortality	Cancer specific mortality	Biochemical recurrence free survival
Wang / 2020[64]	2 009 504 male patients with T2DM, more than 100 000 with PC	24	No association between metformin use and PC risk, however, exploratory analyses suggest that metformin use may be protective in a certain subgroup of patients.	RR = 0.97 (95% CI:0.84-1.12) in case-control and RR = 0.94 (95% CI: 0.79-1.12) in cohort studies							
B Ghiasi / 2019[61]	877 058	11	Not statistically significant reduction of PC risk among metformin users	OR = 0.89 (95% CI: 0.67-1.17)							
Zhaohan / 2019[62]	52 328	18	No association between metformin use and PC risk.	RR = 0.97, (95% CI 0.80-1.16, P = 0.711)							
Chen / 2018[65]	1 572 307 patients in general, 1 171 643 Western and 400 664 Asians	23 - Western 3- Asian	No association between metformin use and risk of PC, in either Western-based or Asian-based populations (after removing a highly influential Asian-based study)	RR = 1.01 (95%CI: 0.86-1.18)							
Wu / 2015[122]	863,769 participants and 39,073 PC	10	No association between metformin use and prostate cancer risk.	RR = 0.92 (95% CI: 0.84-1.02, P = 0.112)							
He / 2019[63]	1,660,795	30	No association between metformin use and PC incidence. Metformin therapy significantly improve OS, CSS, RFS	HR = 0.86 (95% CI: 0.55-1.34, P = 0.51)	HR = 0.72 (95%CI: 0.59-0.88, P = 0.001)	HR = 0.78 (95% CI: 0.64-0.94, P = 0.009)	HR = 0.60 (95% CI: 0.42-0.87, P = 0.006)				
Deng / 2015[60]	334 532	13	Association between metformin use and significantly decreased incidence of PC, however, not with decreased all-cause mortality or decreased recurrence of PC	RR = 0.88 (95% CI: 0.78-0.99, P = 0.03)				RR = 0.90 (95% CI: 0.75-1.09, P = 0.27)	RR = 1.07 (95% CI: 0.86-1.32, P = 0.55)		RR = 0.90 (95% CI 0.75, 1.09, P = 0.27).
Cao X / 2017[123]	37 015 diabetic cancer patients	42	Association between metformin use and reduced ACM and CSM in diabetic patients with PC.						HR = 0.73 (95% CI = 0.68 - 0.79, P < .001)	HR = 0.74 (95% CI = 0.64-0.86, P < .001)	
Stopsack / 2016[69]	9,186 patients	9	Metformin use was associated with improved OS and decreased risk of biochemical recurrence.		HR = 0.88 (95 % CI: 0.86-0.90, p < 0.001)					HR = 0.76 (95 % CI 0.44-1.31, P = 0.33)	HR = 0.79 (95 % CI 0.63-1.00, P = 0.047)
Hwang / 2015[70]	-	8	Metformin use increase risk of cancer recurrence, compared with those who not used metformin. A similar trend was observed for other outcomes, but their relationships did not reach statistical significance.					RR = 1.20 (95%CI: 1.00-1.44)			
Y Xiao / 2017[68]	177,490	13	Metformin user was associated with improved OS and CSS		HR = 0.79 (95% CI: 0.63–0.98) in 8 studies					HR = 0.76 (95% CI: 0.57–1.02) in 6 studies	
Raval / 2015[66]	-	9	Marginally association between metformin use and reduction in the risk of biochemical recurrence. Metformin use was not significantly associated with metastases all-cause mortality and PC -specific mortality						pHR = 0.86 (95% CI : 0.67-1.10, P=0.23, I2: 73%) in 6 studies	pHR= 0.76, (95% CI: 0.43 -1.33, P = 0.33, I2=60%) in 4 studies	pHR = 0.82 (95% CI: 0.67, 1.01, P=0.06, I2=25%) in 5 studies
C.Coyle / 2016[67]	RFS 9330 patients in 6 studies OS 4457 patients in 4 studies CSS 1643 patients in 3 studies		Metformin use demonstrated a borderline significant improvement in RFS , and significant improvements in OS and CSS in PC patients arm		HR = 0.82 (95% CI: 0.73–0.93)		HR = 0.83, (95% CI 0.69–1.00)			HR = 0.58 (95% CI 0.37–0.93)	
